# Factors associated with length of stay following trans-catheter aortic valve replacement - a multicenter study

**DOI:** 10.1186/s12872-017-0573-7

**Published:** 2017-05-26

**Authors:** Yaron Arbel, Nevena Zivkovic, Dhruven Mehta, Sam Radhakrishnan, Stephen E. Fremes, Effat Rezaei, Asim N. Cheema, Sami Al-Nasser, Ariel Finkelstein, Harindra C. Wijeysundera

**Affiliations:** 1 Schulich Heart Centre, Division of Cardiology and Cardiac surgery, Sunnybrook Health Sciences Centre, Toronto, ON Canada; 2 University of Toronto, Toronto, ON Canada; 3 Institute for Clinical Evaluative Sciences (ICES), 2075 Bayview Avenue, Suite A202, Toronto, ON M4N 3M5 Canada; 4 Sunnybrook Research Institute (SRI), Dept. of Medicine & Institute of Health Policy, Management and Evaluation, University of Toronto, Toronto, ON Canada; 5 St. Michael’s Hospital, Toronto, ON Canada; 6 Department of Cardiology, Tel Aviv Medical Center, Tel Aviv, affiliated to the Sackler School of Medicine, Tel Aviv University, Tel Aviv, Israel

**Keywords:** Transcatheter aortic valve replacement, Length of stay, Competing risks, Conscious sedation

## Abstract

**Background:**

Most patients undergoing Transcatheter aortic valve implantation (TAVR) are elderly with significant co-morbidities and there is limited information available regarding factors that influence length of stay (LOS) post-procedure. The aim of this study was to identify the patient, and procedural factors that affect post-TAVR LOS using a contemporary multinational registry.

**Methods:**

We conducted a retrospective cohort study, with patients recruited from three high volume tertiary institutions. The primary outcome was the LOS post-TAVR procedure. We examined patient and procedural factors in a cause-specific Cox multivariable regression model to elucidate their effect on LOS, accounting for the competing risk of post-procedural death. Hazard ratios (HR) greater than 1 indicate a shorter LOS, while HRs less than 1 indicate a longer LOS.

**Results:**

The cohort consisted of 809 patients. Patient factors associated with longer LOS were older age, prior atrial fibrillation, and greater patient urgency. Patient factors associated with shorter LOS were lower NYHA class, higher ejection fraction and higher mean aortic valve gradients.

Procedural characteristics associated with shorter LOS were conscious sedation (HR = 1.19, 95% CI 1.06–1.35, *p* = 0.004). Transapical access was associated with prolonged LOS (HR = 0.49, 95% CI 0.41–0.58, *p* < 0.001).

**Conclusion:**

This multicenter study identified potentially modifiable patient and procedural factors associated with a prolonged LOS. Future research is needed to determine if interventions focused on these factors will translate to a shorter LOS.

**Electronic supplementary material:**

The online version of this article (doi:10.1186/s12872-017-0573-7) contains supplementary material, which is available to authorized users.

## Background

Aortic stenosis (AS) is the most frequent degenerative valvular heart disease in Western countries with a prevalence that has increased in parallel with the aging demographic of the population [1, 2]. AS patients are typically elderly with multiple co-morbidities, which precludes traditional surgical aortic valve replacement (SAVR) [3, 4]. Transcatheter aortic valve replacement (TAVR) has emerged over the last decade as an innovative procedure to deliver aortic valves percutaneously in a minimally invasive manner; based on landmark studies, guidelines now recommend TAVR as the preferred treatment for severe AS patients who are inoperable, and as a reasonable alternative in patients with high operative risk [5]. Emerging evidence supports the expansion of TAVR indications to intermediate risk patients [6]. As such, there has been an exponential growth in the demand for TAVR which will likely increase in the future.

Given the extensive pre-procedural diagnostic work-up, the complexity of the prosthesis and procedure, and the requirement for close post-procedural follow-up, TAVR is extremely resource intensive [7–9]. Given the current economic environment, there is increasing need to improve the efficiency of health care delivery in complex interventions such as TAVR, in order to improve the overall value of health care and also improve equitable access to this life-saving intervention within strict budgetary constraints. One such area specific to TAVR is improving post-procedural length of stay (LOS). In addition to its economic benefits, optimizing LOS in an elderly population has a number of other potential clinical advantages such as reducing infection rates, increasing rehabilitation rates, avoiding malnutrition, and improving overall psychological status.

Although TAVR has been shown to have shorter hospitalizations compared to SAVR [10], some TAVR patients nonetheless have prolonged post-procedural stay [7, 11]. Moreover, there is a wide variation in reports of TAVR LOS reinforcing the likelihood of inefficient care. For example, some studies report the mean hospital stay following TAVR to be 11–13 days [7, 12], while other contemporary cohorts demonstrate hospitalization duration shorter than three days in approximately 30% of patients [11, 13, 14].

There is a paucity of data on the drivers that impact LOS post-TAVR. Elucidating these factors is important so as to identify potentially modifiable factors that can lead to quality improvement interventions to optimize LOS. To address this gap in knowledge, we sought to identify the major drivers of post-TAVR LOS using a contemporary multinational registry.

## Methods

We conducted a retrospective cohort study, with patients recruited from three tertiary institutions, including two in Toronto, Canada (Sites 1 and 3) and one in Tel Aviv, Israel (Site 2). Research ethics board approval was obtained from all centers for the prospective databases. The need for individual patient consent was waived by all the institutional review boards.

### Patient selection

Patients were identified from the TAVR registries from each institution. Each registry collects data on all TAVR patients prospectively. Inclusion criteria were all patients that had a TAVR procedure from January 1st, 2012 to December 31st, 2014. This time frame was chosen in order to include only patients receiving a contemporary TAVR valve procedure, and after each site completed at least 100 cases to overcome the learning curve associated with TAVR. We treated in-hospital death as a competing risk and included patients who died during the procedure or during the TAVR hospitalization.

### Outcome variable

The primary outcome was the LOS post-TAVR procedure. The day of the TAVR was considered time 0, with each 24-h period after this recorded as 1 day. Additional outcomes of interest included peri-procedural minor and major complications, which were defined according to the Valve Academic Research Consortium (VARC- 2) criteria [15].

### Covariates

We also documented the length of time (in days) patients were admitted prior to the TAVR procedure as well as the total LOS, from the date of admission to discharge from the TAVR hospital. For the purpose of analyses, we categorized pre-TAVR admission into 2 groups: a) those admitted electively up to two days prior to the procedure and b) those with a TAVR pre-admission of ≥3 days. This categorization was consistent with these hospitals’ clinical practice based on the advice of our clinical experts, as patients admitted ≥3 days pre-TAVR have pre-procedural clinical deterioration. We determined if the procedure was done 1 day prior to the weekend and contrasted these from procedures done earlier in the week. Patient characteristics that were abstracted from the registries included age, gender, body-mass index (BMI), co-morbidities, as well as mean gradient across the aortic valve.

Peri-procedural risk of in-hospital/30 day mortality was captured by the Society for Thoracic Surgery (STS) score [16, 17]. This score includes approximately 30 variables, including demographics, risk factors, previous interventions, pre-operative status, hemodynamics, and coronary anatomy, and has been well validated to accurately predict peri-operative mortality and morbidity.

Procedural factors included the access type, trans-catheter heart valve type, conscious or general anesthesia, and procedural time. We also included the year of the procedure to account for temporal improvements in care delivery.

### Statistical analysis

Data completeness was verified for all covariates and there were <1% missing data in any co-morbidity field with the exception of EuroScore [18]; as such a complete case analysis was conducted. Descriptive statistics were compared across the 3 hospitals for baseline data, procedure characteristics, and complications, with χ^2^ tests for categorical variables and one-way analysis of variance (ANOVA) for continuous variables.

In order to identify the drivers of LOS, we developed a cause-specific Cox-proportional hazard model, where the dependent variable was the post-TAVR LOS, measured in days [19, 20]. In our study, this is analogous to modeling the rate (or hazard) of being discharged. Hazard ratios (HR) greater than 1 indicate a shorter LOS, while HRs less than 1 indicate a longer LOS. We built a cause-specific Cox model to account for the competing risk of in-hospital death, which precludes the possibility of the primary outcome (discharge). The models included a sandwich type variance estimator to account for clustering of patients at each of the three institutions. We compared the unadjusted LOS between the 3 hospitals using this model.

Prior to inclusion into the final covariate-adjusted model, we assessed all baseline factors for co-linearity based on the variance inflation factor but found no important multi-colinearities. To build the final covariate adjusted model, we planned to include all variables except for EuroScore due to >5% missing data. The results for creatinine lacked face validity in the adjusted model so it was excluded. All other variables were included as this was an explanatory model. The proportional hazards assumption was tested by computing Martingale residuals for the final model.

A sensitivity analysis was conducted to understand the role of complications in TAVR post-procedural LOS. For each complication, we calculated both an unadjusted HR and one adjusted for all co-variates in the full model. All of the analyses were considered significant at a two-tailed *p*-value of less than 0.05. All analysis was done with SAS 9.3.

## Results

**Fig. 1:**
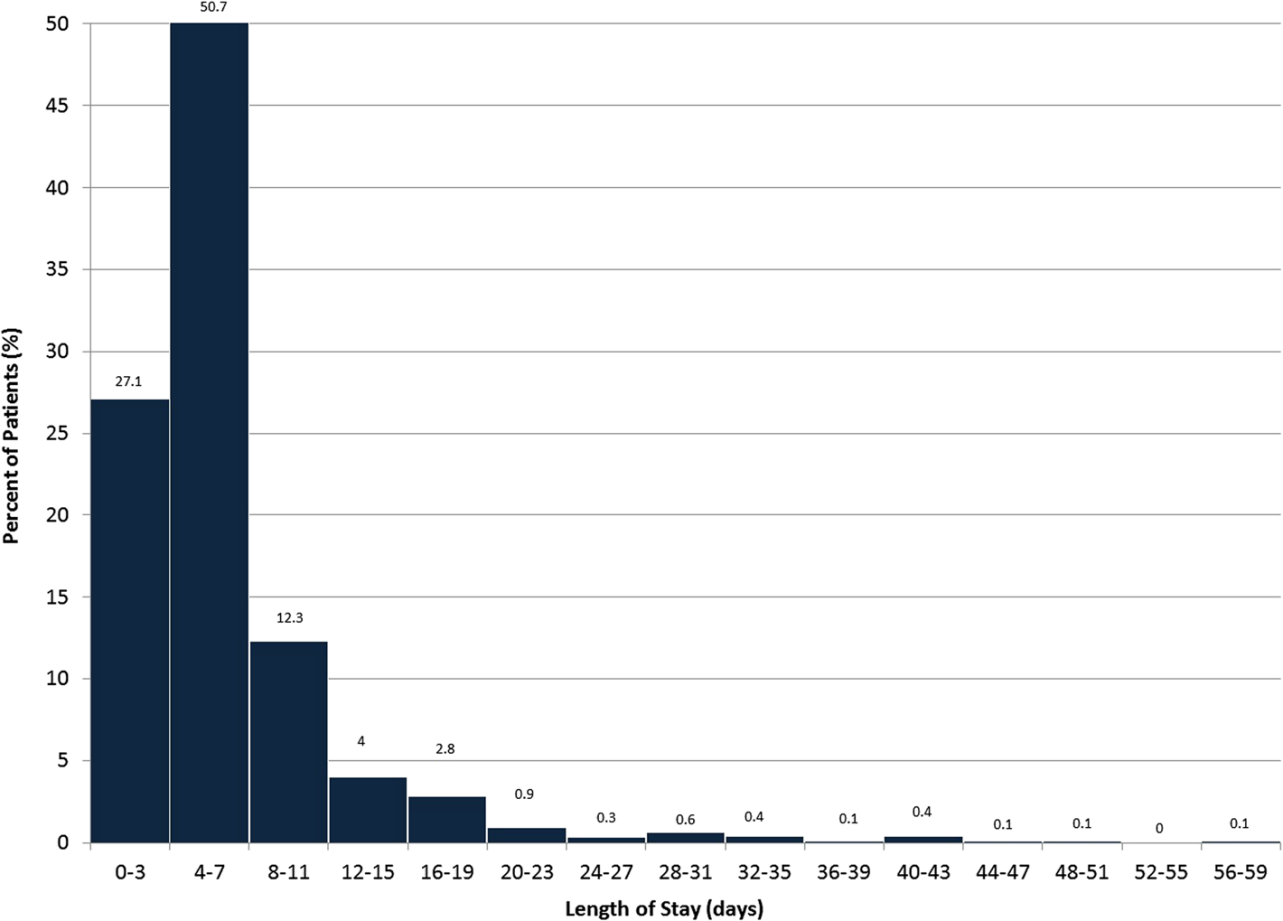
Distribution of Post-TAVR Length of Stay of Discharged Patients. Figure shows percentage of patients discharged in each time period following TAVR. All patients have a minimum LOS of 2 days

We included a total of 809 patients from three centers over the time period of interest. Mean pre-TAVR LOS differed between the three sites. Site two routinely admitted elective patients one to two days before TAVR, while the other sites generally admitted these patients on the morning of the procedure (Table 1). Figure 1 demonstrates the post-TAVR LOS. Approximately 27.1% of patients had post-procedural LOS between 2 to 3 days. The three sites show no significant differences in terms of post-TAVR or total LOS. The mean post-procedural LOS was 6.6 days, with means of 6.9, 5.8 and 7.7 at the three sites, while mean total LOS was calculated at 8.8 days across sites, with site-specific values of 9.8, 7.9 and 9.4, respectively.

**Table 1:** Baseline Patient and Procedural Characteristics. Cohort baseline and procedural characteristics according to site. All covariates are presented as frequencies and percentages, with the exception of mean values. Mean values include standard deviation

	Total (*N* = 809)	Site 1 (*n* = 164)	Site 2 (*n* = 406)	Site 3 (*n* = 239)	*p* Value
Patient Characteristics					
Age (years)	82.7 ± 6.4	83.7 ± 6.4	82.4 ± 6.2	82.6 ± 6.7	0.095
Female	415 (51.3%)	79 (48.2%)	219 (53.9%)	117 (49.0%)	0.316
BMI	27.1 ± 5.3	27.0 ± 6.1	27.3 ± 5.3	26.6 ± 4.6	0.224
STS	6.0 ± 5.4	4.4 ± 2.8	4.3 ± 2.9	10.0 ± 7.6	<0.001
NYHA					<0.001
1	2 (0.3%)	2 (1.2%)	0	0	
2	99 (12.3%)	15 (9.2%)	55 (13.6%)	29 (12.3%)	
3	505 (62.9%)	120 (73.6%)	227 (56.2%)	158 (67.0%)	
4	197 (24.5%)	26 (16.0%)	122 (30.2%)	49 (20.8%)	
Atrial Fibrillation	280 (34.6%)	68 (41.5%)	135 (33.3%)	77 (32.2%)	0.114
Hypertension	717 (88.6%)	141 (86.0%)	358 (88.2%)	218 (91.2%)	0.245
Diabetes Mellitus	279 (34.5%)	48 (29.3%)	155 (38.2%)	76 (31.8%)	0.075
Dyslipidemia	645 (79.8%)	114 (69.5%)	333 (82.2%)	198 (82.9%)	0.001
COPD	129 (16.0%)	16 (9.8%)	55 (13.6%)	58 (24.3%)	<0.001
Coronary Artery Disease	520 (64.3%)	87 (53.1%)	260 (64.0%)	173 (72.4%)	<0.001
Peripheral Vascular Disease	93 (11.5%)	24 (14.6%)	17 (4.2%)	52 (21.8%)	<0.001
Dialysis	26 (3.2%)	5 (3.1%)	10 (2.5%)	11 (4.6%)	0.328
Permanent Pacemaker	101 (12.5%)	25 (15.2%)	55 (13.6%)	21 (8.8%)	0.103
Prior Stroke	120 (14.8%)	35 (21.3%)	52 (12.8%)	33 (13.8%)	0.030
Prior Cardiac Surgery	203 (25.1%)	46 (28.1%)	89 (21.9%)	68 (28.5%)	0.113
Prior PCI	196 (24.2%)	32 (19.5%)	55 (13.6%)	109 (45.6%)	<0.001
Pre-Procedural Ejection Fraction (%)	54.3 ± 10.7	51.7 ± 9.0	55.1 ± 8.6	54.5 ± 14.3	0.003
Pre-Procedural Mean Pressure Gradient (mmHg)	46.4 ± 15.9	48.8 ± 16.3	46.2 ± 15.0	45.2 ± 16.8	0.071
Pre-Procedural Creatinine (μmol/L)	107.9 ± 64.0	113.6 ± 88.9	104.0 ± 58.9	110.6 ± 50.5	0.203
Procedural Characteristics					
Procedure Year					0.015
2012	224 (27.7%)	42 (25.6%)	129 (31.8%)	53 (22.2%)	
2013	274 (33.9%)	66 (40.2%)	131 (32.3%)	77 (32.2%)	
2014	311 (38.4%)	56 (34.2%)	146 (36.0%)	109 (45.6%)	
Procedure Prior to Weekend^a^	185 (22.9%)	2 (1.2%)	26 (6.4%)	157 (65.7%)	<0.001
Procedure Time (min)	161.1 ± 67.5	87.4 ± 38.8	168.9 ± 50.1	199.6 ± 69.7	<0.001
Access Site					<0.001
Transfemoral	737 (91.1%)	111 (67.7%)	405 (99.8%)	221 (92.5%)	
Subclavian	2 (0.3%)	0	1 (0.3%)	1 (0.4%)	
Suprasternal	1 (0.1%)	1 (0.6%)	0	0	
Direct Aortic	26 (3.2%)	10 (6.1%)	0	16 (6.7%)	
Transapical	43 (5.3%)	42 (25.6%)	0	1 (0.4%)	
Anesthesia Type					<0.001
General Anesthesia	408 (50.4%)	164 (100%)	5 (1.2%)	239 (100%)	
Local/Conscious Sedation	401 (49.6%)	0	401 (98.8%)	0	
Valve Used					<0.001
Medtronic CoreValve or Evolut R	458 (56.7%)	1 (0.6%)	253 (62.6%)	204 (85.3%)	
Edwards XT or S3	332 (41.2%)	155 (94.5%)	146 (36.1%)	31 (13%)	
Portico	17 (0.2%)	8 (4.9%)	5 (1.2%)	4 (1.7%)	
Valve in Valve	34 (4.2%)	8 (4.9%)	0	26 (10.9%)	<0.001
Pre-TAVR Admission					<0.001
0 days	305 (37.7%)	115 (70.12%)	1 (0.3%)	189 (79.1%)	
1–2 days	415 (51.3%)	14 (8.54%)	382 (94.1%)	19 (8%)	
≥ 3 days	89 (11%)	35 (21.34%)	23 (5.7%)	31 (13%)	

*BMI* Body Mass Index, *COPD* Chronic Obstructive Pulmonary Disease, *NYHA* New York Heart Association functional classification, *PCI* percutaneous coronary intervention, *STS* Society of Thoracic Surgeons adult cardiac surgery risk score

^a^Refers to procedures that take place one day prior to the weekend

The incidence of complications for the entire cohort is summarized in Additional file 1: Table S1, as is post-procedure aortic regurgitation and post-procedure mitral regurgitation. Thirty patients (3.7%) died in hospital, while 30-day mortality was 4.9%. The rates of complications were comparable to those found in the literature, with 18 (2.2%) having a stroke, 107 (13.2%) requiring a permanent pacemaker, 49 (6.1%) suffering from a major vascular complication, and 37 (4.6%) having major bleeding.

As seen in Table 2, there was a temporal effect on LOS identified in the multivariable models, with patients undergoing TAVR in 2012 having a longer LOS compared to those in 2014. We found the following patient characteristics to be associated with longer length of stay: prior atrial fibrillation (HR = 0.76, 95% CI 0.69–0.84, *p* < 0.001), each one-year increase in patient age (HR = 0.990, 95% CI 0.985–0.995, *p* < 0.001), and patient urgency as indicated by pre-TAVR admission of three or more days (HR = 0.64, 95% CI 0.46–0.90, *p* = 0.009).

**Table 2:** Relationship between Baseline and Procedural Characteristics with Post-TAVR Length of Stay, Adjusted by Model Covariates. Multivariable analysis of the variables associated with length of stay

Parameter	Hazard Ratio (95% Confidence Interval)	*p* Value
Year		
2014	Referent	
2012	0.78 (0.68–0.9)	<0.001
2013	0.85 (0.73–0.98)	0.021
Sex		
Female	Referent	
Male	1.12 (0.97–1.3)	0.133
Age	0.99 (0.99–1)	<0.001
Pre-TAVR Admission		
0–2 days	Referent	
≥ 3 days	0.64 (0.46–0.9)	0.009
BMI	1 (0.98–1.02)	0.920
STS Risk Score	0.98 (0.96–1)	0.064
NYHA Class		
4	Referent	
1–2	1.28 (1.1–1.49)	0.001
3	1.16 (1.03–1.31)	0.019
Atrial Fibrillation	0.76 (0.69–0.84)	<0.001
Hypertension	1 (0.83–1.2)	0.960
Diabetes Mellitus	1 (0.89–1.13)	0.986
Dyslipidemia	0.92 (0.68–1.24)	0.577
Coronary Artery Disease	0.98 (0.82–1.16)	0.805
Prior PCI	1.05 (0.84–1.31)	0.670
Prior Open Heart Surgery	1.15 (1.09–1.21)	<0.001
Dialysis	0.95 (0.64–1.41)	0.797
COPD	0.94 (0.75–1.17)	0.558
Peripheral Vascular Disease	1.01 (0.81–1.25)	0.948
Permanent Pacemaker	1.1 (0.74–1.62)	0.644
Stroke	0.81 (0.58–1.13)	0.215
Pre-Procedural Ejection Fraction (1ss0%)	1.05 (1.03–1.07)	<0.001
Pre-Procedural Mean Pressure Gradient	1 (1–1.01)	<0.001
Procedure Time (10 min)	0.98 (0.96–0.99)	0.001
Access Site		
Transfemoral	Referent	
Direct Aortic	0.76 (0.67–0.85)	<0.001
Subclavian or Suprasternal	2.42 (1.4–4.2)	0.002
Transapical	0.49 (0.41–0.58)	<0.001
Anesthesia Type		
General Anesthesia	Referent	
Local/Conscious Sedation	1.19 (1.06–1.35)	0.004
Valve Used		
Medtronic CoreValve or Evolut-R	Referent	
Edwards XT or S3	0.99 (0.96–1.03)	0.647
Portico	0.43 (0.25–0.73)	0.002
Valve in Valve Procedure	1.28 (1.15–1.41)	<0.001
Procedure prior to Weekend^a^	0.88 (0.77–1.01)	0.061

Hazard ratios above 1 indicate shorter time to discharge and shorter length of stay, whereas the opposite is true for ratios below 1. *STS* Society of Thoracic Surgeons adult cardiac surgery risk score, *NYHA* New York Heart Association functional classification, *COPD* chronic obstructive pulmonary disease, *PCI* percutaneous coronary intervention

^a^Refers to procedures that take place one day prior to the weekend

Patients were discharged sooner if they were NYHA class 1 or 2 (HR = 1.28, 95% CI 1.10–1.49, *p* = 0.001) or NYHA class 3 (HR =1.16, 95% CI 1.03–1.31, *p* = 0.019) compared to NYHA class 4. Every 10% increase in ejection fraction was associated with shorter length of stay (HR = 1.05, 95% CI 1.03–1.07, *p* < 0.001), as was each one mmHg increase in the pre-procedural mean aortic valve pressure gradient (HR = 1.004, 95% CI 1.002–1.01, *p* < 0.001). Patients who had at least one prior open heart surgery were also discharged sooner (HR = 1.15, 95%CI 1.09–1.21, *p* < 0.001).

Procedure factors had important impact on post-TAVR LOS. Patients receiving conscious sedation, rather than general anesthesia, had a significantly shorter LOS (HR = 1.19, 95% CI 1.06–1.35, *p* = 0.004). Compared to patients receiving TAVR via trans-femoral approach, direct aortic approach was associated with longer LOS (HR = 0.76, 95% CI 0.67–0.85, *p* < 0.001), as was trans-apical approach (HR = 0.49, 95% CI 0.41–0.58, *p* < 0.001). A subclavian or suprasternal approach was associated with earlier discharge compared to transfemoral (HR = 2.42, 95% CI 1.40–4.20, *p* = 0.002); however, there were few patients with these alternative access sites (*n* = 3) in our cohort. Longer TAVR procedure duration was associated with longer LOS (HR = 0.98 for every 10 min increase, 95% CI 0.96–0.99, *p* = 0.001). In terms of valve type, implantation of a Portico trans-catheter heart valve was associated with longer LOS (HR = 0.43, 95% CI 0.25–0.73, *p* < 0.001). A valve in valve procedure was associated with shorter LOS (HR = 1.28, 95% CI 1.15–1.41, *p* < 0.001).

The unadjusted HR suggest that almost all post procedure complications are statistically associated with longer LOS, except for new arrhythmia and myocardial infarction (*p* = 0.090, *p* = 0.055, respectively). When adjusted for baseline characteristics, all post-procedural complications were associated with a statistically significant increase in length of stay (Table 3).

**Table 3:** Relationship between Presence of Complication to Post-TAVR Length of Stay, with Unadjusted and Adjusted Analyses. Hazard ratios with 95% confidence intervals for post-TAVR LOS according to presence of different complications. Includes unadjusted analysis as well as multivariable adjustment for baseline and procedural characteristics

	Unadjusted Hazard Ratio	*p* Value	Adjusted Hazard Ratio	*p* Value
Vascular				
Major	0.46 (0.33–0.64)	<0.001	0.42 (0.37–0.49)	<0.001
Minor	0.79 (0.64–0.97)	0.023	0.73 (0.58–0.93)	0.011
Stroke	0.31 (0.19–0.50)	<0.001	0.35 (0.22–0.55)	<0.001
MI	0.33 (0.11–1.02)	0.055	0.6 (0.42–0.84)	0.003
Arrhythmia	0.88 (0.76–1.02)	0.090	0.75 (0.61–0.93)	0.008
Permanent Pacemaker	0.59 (0.48–0.73)	<0.001	0.49 (0.37–0.64)	<0.001
Bleeding				
Life Threatening	0.30 (0.17–0.55)	<0.001	0.28 (0.17–0.46)	<0.001
Major	0.54 (0.38–0.76)	<0.001	0.56 (0.46–0.68)	<0.001
Minor	0.69 (0.49–0.98)	0.036	0.82 (0.7–0.95)	0.009
Transfusion	0.56 (0.46–0.68)	<0.001	0.59 (0.54–0.65)	<0.001
Cardio-pulmonary bypass	0.32 (0.12–0.87)	0.025	0.27 (0.18–0.41)	<0.001
Emergency operation	0.31 (0.15–0.65)	0.002	0.31 (0.11–0.91)	0.033
Open heart surgery	0.29 (0.09–0.89)	0.030	0.27 (0.18–0.41)	<0.001
Any Major Complication^a^	0.44 (0.37–0.52)	<0.001	0.38 (0.28–0.5)	<0.001

Hazard ratios >1 indicate shorter time to discharge, while hazard ratios <1 indicate prolonged time to discharge. *MI* myocardial infarction

^a^Patient with any of the following complications: major vascular, stroke, MI, new permanent pacemaker, life-threatening bleeding, major bleeding, cardio-pulmonary bypass, emergency operation, or open heart surgery

## Discussion

In this multi-center study of contemporary TAVR procedures, we found a wide variation in post-TAVR LOS. We identified a number of potentially modifiable factors that influenced LOS, such as access route, type of anesthesia, the timing of the procedure prior to the weekend, procedure time, and valve type. These are potential targets for quality improvement initiatives. Importantly, we found that post-procedural complications were strongly associated with prolonged stay.

Improving efficiency of health care delivery is of particular importance in the current era of budgetary constraints such that scarce health care resources are used optimally. One target for improving efficiency is reduction of LOS. Indeed, there is an abundant literature on LOS in different areas of medicine, specifically the drivers of prolonged LOS as well as strategies to improve it. For example, malnutrition measured by recent weight loss or a BMI below 20 has been associated with prolonged LOS [21]. Surgical site infections are a major driver of LOS in the surgical literature with a doubling of the LOS [22]. Early interventions and internal fixations have been associated with reduced LOS in patients with hip fractures [23]. Improving patients’ renal function [24] as well as pulmonary function have been associated with shorter LOS in patients undergoing cardiac surgery [25]. Increasing hospital volume has been associated with a reduced LOS [26]. Many of these factors are potentially modifiable and thus targets for quality improvement.

TAVR is an example of a complex intervention, targeted to an elderly patient population, often with multiple co-morbidities. Moreover, it is extremely resource intensive, and in many jurisdictions, limited to select tertiary hospitals, each with limited funding. These supply factors, combined with an exponentially growing demand, in particular as indications expand beyond only inoperable and very high risk patients, make TAVR an especially relevant area to identify potential targets for improving efficiency. The limited data on TAVR LOS currently suggests that there is variation in LOS [13, 27, 28].

In our study, we confirmed this wide variation in LOS, extending from 2 days to 59 days. We were able to identify a number of baseline patient characteristics that were associated with prolonged LOS, including prior atrial fibrillation, age, and lower baseline ejection fraction. Indeed, LOS appears to be a surrogate for the burden of co-morbidity. Although these factors are non-modifiable, they nonetheless should alert health care providers of the potential for a longer LOS, and as such, pre-emptive measures could be put into place early to facilitate transitions to the community or in-patient rehabilitation.

We also identified a number of potentially modifiable procedural factors such as procedure done before the weekend, valve type and conscious sedation. Indeed, other groups are evaluating the use of conscious sedation in combination with a care pathway to facilitate next day discharge post TAVR [29–31]. Conscious Sedation and non- surgical access have been associated with less delirium and might explain the shorter LOS. [32] The relationship between length of procedure and higher NYHA with LOS might be explained by the increased incidence of complications in sicker patients. Similarly, in hospital TAVI patients are more frail and await rehabilitation and hence have longer LOS. [33] The relationship between Portico valve and LOS might be attributed to a learning curve since it is a newer valve compared to Corevalve and Sapien valves. Longer LOS when the TAVR procedure is performed prior to the weekend has face validity and may be related to delays in obtaining post-procedural echocardiograms during the weekend, or being able to put into place the necessary transition supports such as homecare. If so, this suggests that development of strategies to mitigate these delays; one such strategy could be performing echocardiography immediately post procedure. These are all potential areas for future study.

It is important to note the complex relationship between pre-procedural LOS and post-procedural hospitalization duration. We grouped patients admitted to hospital 0–2 days pre-TAVR as they likely received an elective TAVR. It is clear that elective pre-admission did not impact post-TAVR LOS. In contrast, patients who were admitted for longer periods prior to their TAVR likely represent patients who had decompensated heart failure. This group has a substantially longer LOS; this illustrates that once TAVR patients deteriorate while waiting for their procedure, this translates into a slower recovery post-procedure. This reinforces the importance of diligent wait-time management for TAVR patients.

In addition, there are some general measures that have been shown to help reduce LOS which might also be relevant to TAVR patients. Patients with expected long LOS might benefit from preemptive interventions [34, 35] including geriatric care and pre-procedural rehab [36, 37], nutrition supplementation [38], reducing bleeding risk [39], optimize periprocedural practice [29, 40, 41], and post procedural rehabilitation [42].

Our study provides a number of new insights to the literature. First, we used competing risk models to include all TAVR patients in our analyses, not only those who survived to discharge. This eliminates the potential biases introduced by only studying patients selected for survival. In addition, we have shown that in TAVR the occurrence of a post-procedural complication has a remarkable influence on LOS and one can hypothesize that the occurrence of complications may be one of the causal pathways by which the factors identified in our models impact LOS. The extended LOS likely explains in part the increased costs associated with complications [8, 39].

Our study must be interpreted in the context of several limitations that merit discussion. Although we had a relatively larger sample size across two countries, and used a clustered model to account for potential site related factors, our cohort was limited to three tertiary care hospitals that had wide variability in clinical practice (access site, sedation type, etc.), which impacts the generalizability of our findings. Second, although we identified factors associated with LOS, it does not follow necessarily that changing those factors will translate to shorter LOS. Instead, potential strategies impacting those factors must be empirically tested. Third, we did not have data on frailty or social status. The number of portico cases was small as were subclavian and direct aortic access routes and therefore, any conclusions regarding these variables may be explained by the learning curve. Finally, our study was an observational study, and there may have residual confounders that we did not account for. As such, our results should be considered hypothesis generating and not conclusive.

## Conclusions

In conclusion, this multicenter study identified a number of important modifiable patient and procedural factors associated with LOS. Future studies should evaluate if interventions focused on these potentially modifiable factors will translate to shorter LOS.

## Additional file

Additional file 1: Table S1. Post-TAVR complications and select echo measurements of study cohort. (DOC 65 kb)

## Abbreviations

AS: Aortic Stenosis
CI: Confidence Interval
HR: Hazard ratio
LOS: Length of stay
NYHA: New York Heart association
STS: Society of Thoracic surgeons
TAVR: Transcatheter aortic valve replacement
