# Supplementary material for: Factors associated with length of stay following trans-catheter aortic valve replacement - a multicenter study
Source: BMC Cardiovasc Disord. 2017 May 26;17:137. doi: 10.1186/s12872-017-0573-7 (PMC5446678; doi:10.1186/s12872-017-0573-7)
Supplement: Additional file 1: Table S1. — Post-TAVR complications and select echo measurements of study cohort. (DOC 65 kb) [file 12872_2017_573_MOESM1_ESM.doc]

| **Table S1.** | | | | | |
| --- | --- | --- | --- | --- | --- |
|  | | | | | |
|  | **Total (N=809)** | **Site 1 (n=164)** | **Site 2 (n=406)** | **site 3 (n=239)** | **P value** |
| **COMPLICATIONS** | | | | | |
| **Death** | 30 (3.71%) | 8 (4.88%) | 15 (3.69%) | 7 (2.92%) | 0.596 |
| **30 Day Death** | 40 (4.94%) | 10 (6.1%) | 15 (3.69%) | 15 (6.28%) | 0.257 |
| **Vascular** |  |  |  |  |  |
| Major | 49 (6.1%) | 7 (4.27%) | 34 (8.37%) | 8 (3.43%) | 0.023 |
| Minor | 109 (13.57%) | 8 (4.88%) | 57 (14.04%) | 44 (18.88%) | <0.001 |
| **Stroke** | 18 (2.22%) | 0 (0%) | 7 (1.72%) | 11 (4.6%) | 0.006 |
| **MI** | 3 (0.37%) | 0 (0%) | 0 (0%) | 3 (1.3%) | 0.024 |
| **Arrythmia** | 320 (40.1%) | 17 (10.37%) | 225 (55.42%) | 78 (34.21%) | <0.001 |
| **Permanent Pacemaker** | 107 (13.23%) | 4 (2.44%) | 63 (15.52%) | 40 (16.74%) | <0.001 |
| **Bleeding** |  |  |  |  |  |
| Life Threatening | 25 (3.11%) | 3 (1.83%) | 5 (2.14%) | 5 (2.14%) | 0.203 |
| Major | 37 (4.6%) | 4 (2.44%) | 23 (5.67%) | 10 (4.27%) | 0.241 |
| Minor | 33 (4.1%) | 8 (4.88%) | 0 (0%) | 25 (10.68%) | <0.001 |
| **Transfusion** | 145 (18.17%) | 11 (6.71%) | 90 (22.56%) | 44 (18.72%) | <0.001 |
| **Cadio-pulmonary bypass** | 8 (1%) | 1 (0.61%) | 2 (0.49%) | 5 (2.16%) | 0.108 |
| **Emergency operation** | 12 (1.49%) | 0 (0%) | 5 (1.23%) | 7 (2.93%) | 0.049 |
| **Open heart surgery** | 6 (0.74%) | 1 (0.61%) | 2 (0.49%) | 3 (1.26%) | 0.539 |
| **Aortic Regurgitation** |  |  |  |  | <0.001 |
| 0 | 216 (26.97%) | 51 (31.88%) | 128 (31.53%) | 37 (15.74%) |  |
| 1 | 347 (43.32%) | 47 (29.38%) | 192 (47.29%) | 108 (45.96%) |  |
| 2 | 192 (23.97%) | 51 (31.88%) | 56 (13.79%) | 85 (36.17%) |  |
| 3 | 43 (5.37%) | 11 (6.88%) | 27 (6.65%) | 5 (2.13%) |  |
| 4 | 3 (0.37%) | 0 (0%) | 3 (0.74%) | 0 (0%) |  |
| **Mitral Regurgitation** |  |  |  |  | <0.001 |
| 0 | 57 (7.15%) | 5 (3.18%) | 43 (10.59%) | 9 (3.85%) |  |
| 1 | 246 (30.87%) | 57 (36.31%) | 104 (25.62%) | 85 (36.32%) |  |
| 2 | 355 (44.54%) | 61 (38.85%) | 182 (44.83%) | 112 (47.86%) |  |
| 3 | 134 (16.81%) | 34 (21.66%) | 74 (18.23%) | 26 (11.11%) |  |
| 4 | 5 (0.63%) | 0 (0%) | 3 (0.74%) | 2 (0.85%) |  |
| **Any major complication** | 190 (23.49%) | 18 (10.98%) | 107 (26.35%) | 65 (27.2%) | <0.001 |

**Table S1**- Post-TAVR complications and select echo measurements of study cohort according to site. Data is presented as frequencies and percentages to describe prevalence of complication, with exception of variables with mean values. Mean values include standard deviation.

Note: MI = myocardial infarction.

*Patient with any of the following complications: major vascular, stroke, MI, new permanent pacemaker, life-threatening bleeding, major bleeding, cardio-pulmonary bypass, emergency operation or open heart surgery.
